# Understanding of ROS-Inducing Strategy in Anticancer Therapy

**DOI:** 10.1155/2019/5381692

**Published:** 2019-12-18

**Authors:** Su Ji Kim, Hyun Soo Kim, Young Rok Seo

**Affiliations:** ^1^Institute of Environmental Medicine for Green Chemistry, Dongguk University Biomedi Campus, 32 Dongguk-ro, Ilsandong-gu, Goyang-si, Gyeonggi-do 10326, Republic of Korea; ^2^Department of Life Science, Dongguk University Biomedi Campus, 32 Dongguk-ro, Ilsandong-gu, Goyang-si, Gyeonggi-do 10326, Republic of Korea

## Abstract

Redox homeostasis is essential for the maintenance of diverse cellular processes. Cancer cells have higher levels of reactive oxygen species (ROS) than normal cells as a result of hypermetabolism, but the redox balance is maintained in cancer cells due to their marked antioxidant capacity. Recently, anticancer therapies that induce oxidative stress by increasing ROS and/or inhibiting antioxidant processes have received significant attention. The acceleration of accumulative ROS disrupts redox homeostasis and causes severe damage in cancer cells. In this review, we describe ROS-inducing cancer therapy and the anticancer mechanism employed by prooxidative agents. To understand the comprehensive biological response to certain prooxidative anticancer drugs such as 2-methoxyestradiol, buthionine sulfoximine, cisplatin, doxorubicin, imexon, and motexafin gadolinium, we propose and visualize the drug-gene, drug-cell process, and drug-disease interactions involved in oxidative stress induction and antioxidant process inhibition as well as specific side effects of these drugs using pathway analysis with a big data-based text-mining approach. Our review will be helpful to improve the therapeutic effects of anticancer drugs by providing information about biological changes that occur in response to prooxidants. For future directions, there is still a need for pharmacogenomic studies on prooxidative agents as well as the molecular mechanisms underlying the effects of the prooxidants and/or antioxidant-inhibitor agents for effective anticancer therapy through selective killing of cancer cells.

## 1. Introduction

Reactive oxygen species (ROS) are generally defined as chemically reactive molecules containing oxygen, produced as a result of cellular metabolism [1]. A moderate level of ROS plays an essential role in the cellular signaling that regulates cell proliferation and cell survival [2]. However, an increase in ROS levels can damage cellular components such as lipids, proteins, and DNA, causing an imbalance between cellular reduction-oxidation (redox) conditions and resulting in the disruption of homeostasis [3]. Chronically increased ROS cause severe cellular damage and lead to carcinogenesis by modulating cell signaling in biological processes including cell proliferation and survival, angiogenesis, and metastasis [4, 5].

**Figure 1:**
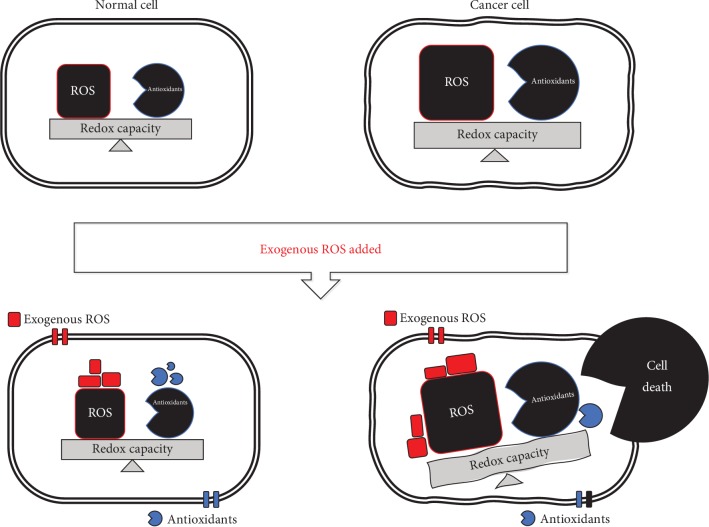
Differential ROS levels in normal and cancer cells. Normal cells have a lower basal ROS level than cancer cells. In normal cells, a moderate ROS level is essential to promote cell proliferation and survival whereas an excessive ROS level has detrimental effects such as tumor progression and angiogenesis. The redox balance in cancer cells is readily regulated by increasing antioxidant processes. Once the ROS level exceeds the redox capacity in cancer cells, severe oxidative stress occurs, resulting in cancer cell death via the activation of apoptosis, autophagic cell death, and necroptosis.

Anticancer therapies based on oxidative damage through the acceleration of accumulative ROS or the defective antioxidant system in cancer cells have been developed [2, 6]. Due to uncontrolled metabolic processes during hyperproliferation, cancer cells have a higher basal ROS level than normal cells [7]. Adaptation to excessive ROS conditions in cancer cells has been reported, suggesting they have a higher level of antioxidative capacity and ROS than normal cells [2]. ROS-inducing approaches rely on the fact that increasing the ROS level over the cytotoxic threshold can selectively kill cancer cells. The elevated ROS level breaks the redox homeostasis and consequently causes cancer cell death. If exogenous ROS-generating agents are triggered, the redox-imbalanced cancer cells become more vulnerable than normal cells, thereby leading to cell death [8] (Figure 1). Accordingly, prooxidative agents have been investigated as anticancer drugs that interrupt redox adaptation and eventually induce cytotoxicity in ROS-dependent cancer cells [9].

In this review, we summarize the mechanisms underlying the effects of anticancer drugs utilized in oxidative stress-inducing chemotherapy for direct or indirect ROS generation. To grasp the biological alterations mediated by prooxidative drugs, the drug-focused pathways were analyzed and visualized using big data-based network analysis software. We also suggest crucial therapeutic strategies for anticancer drugs and provide information regarding potential side effects and drug resistance based on the results of the pathway analysis.

## 2. Basic Concepts of ROS: Generation and Elimination

Oxygen is an essential molecule for maintaining metabolism and life in organisms. However, the metabolism of oxygen produces highly reactive molecules called ROS, a major source of oxidative stress. There are many types of ROS, including superoxide (O_2_^·-^), hydroxyl radicals (OH^·^), hydrogen peroxide (H_2_O_2_), and singlet oxygen (^1^O_2_) [10]. The cellular redox state refers to the balance between the oxidized and reduced states in cells. In living organisms, redox equilibrium is important for cellular homeostasis [11]. As previously demonstrated, the impairment of redox homeostasis mediated by an excess of oxidized biological molecules is associated with cellular toxic effects [12]. Accordingly, proper regulation of the redox status through ROS generation and elimination is crucial.

Most endogenous ROS are mainly generated in the mitochondrial electron transport chain (ETC) and NADPH oxidase complex (NOX) [13, 14]. During oxidative phosphorylation, the leakage of electrons by ETC complexes I and III occurs in the inner mitochondrial membrane, leading to the reduction of oxygen into superoxide. Subsequently, superoxide dismutase (SOD) converts superoxide into hydrogen peroxide in the intermembrane space or the matrix of mitochondria [8, 14]. Hydrogen peroxide can be converted into hydroxyl radicals in the presence of Fe^2+^ [15]. Likewise, NOX, a transmembrane enzyme complex consisting of seven subunits, catalyzes the oxidation of NADPH by transferring electrons to molecular oxygen, leading to the production of superoxide [16].

To avoid endogenous ROS overproduction, cells have diverse defense systems to eliminate ROS using antioxidant molecules and enzymes such as glutathione (GSH), peroxiredoxin (Prx), thioredoxin (Trx), SOD, and catalase [17]. GSH protects cellular components against oxidative damage through interactions with a cofactor of GSH peroxidase (GPx) and/or participation in other antioxidant components [18, 19]. In the presence of NADPH, GSH reductase catalyzes the reduction of GSH. Two reduced GSH molecules are oxidized into GSH disulfide (GSSG) via a reaction with GPx, which catalyzes the reduction of hydrogen peroxide to water and oxygen molecules through the redox cycle [20, 21]. GSH deficiency has been shown to reduce tissue ascorbate levels and increase oxidative stress, ultimately resulting in diverse disorders such as mitochondrial disease, hepatic injuries, and HIV [19, 22, 23]. Several anticancer drugs and xenobiotics have been developed for GSH-targeted chemotherapies or detoxifying agent-based chemoprevention [24]. Both Prx and Trx, which contain cysteine residues with redox-reactive thiol groups, can scavenge hydrogen peroxide via thiol/disulfide exchange [25]. Hydrogen peroxide is reduced by Prx, which is simultaneously oxidized to form a disulfide bond, and Prx is subsequently reduced by transferring the disulfide bond to Trx [26]. In the presence of NADPH, Trx is reduced by a reaction with Trx reductase [27, 28]. SOD catalyzes the breakdown of superoxide to molecular oxygen and hydrogen peroxide using metal ion cofactors including copper, zinc, and manganese [29, 30]. Catalases reduce hydrogen peroxide to water and oxygen with a manganese ion cofactor [31].

Although cellular antioxidant systems have a vital role in balancing endogenous ROS levels and the redox status for cell protection against oxidative stress [32, 33], exogenously prooxidants-induced ROS levels and an ineffective cellular defense system result in significant imbalance between prooxidants and antioxidants [34], possibly enabling cellular damage and cell death.

## 3. Application of ROS Induction for Anticancer Strategies

A lot of anticancer therapies have employed antioxidant supplements as a strategy to prevent or treat cancer cells. *tert*-Butylhydroquinone (tBHQ) mediates the dissociation of Nrf2 via oxidative modification of the Keap1 cysteine residues by ROS generated during the metabolic process [35]. Nrf2 activation promotes the regulation of downstream cytoprotective genes, which play important roles in cancer prevention [36]. Selenocompounds exhibit anticancer effects through potentiating the antioxidative defense system from ROS-induced cellular damage [37, 38] and through redox modification of redox-active, cysteine-rich regions of protein kinase C (PKC), a receptor for tumor promoters [39, 40].

However, controversial issues remain regarding the chemotherapeutic activities of antioxidants. Indeed, it has been widely reported that Nrf2 activation contributes to chemoresistance in cancer cells [41–44]. Additionally, a high concentration of tBHQ has been reported to increase carcinogenic risk [45, 46]. The efficacy and safety of selenium are also actively discussed due to its toxicity and side effects [47, 48]. Thus, chemotherapies involving antioxidants may not be sufficient to kill cancer cells and further studies are needed to determine whether they have unexpected adverse effects.

ROS has double-edged sword characteristics in terms of its low-dose cell signaling and high-dose cytotoxicity [49]. A mild level of ROS regulates cell development and homeostasis, whereas a high level inflicts severe cellular damage [50, 51]. Cancer cells are more sensitive to the presence of prooxidants and the inhibition of antioxidants due to their excessive ROS levels [52–54]. The ROS-inducing approach for killing cancer cells relies on oxidative stress-dependent cytotoxic effects through apoptosis, necroptosis, and autophagic cell death [55].

**Figure 2:**
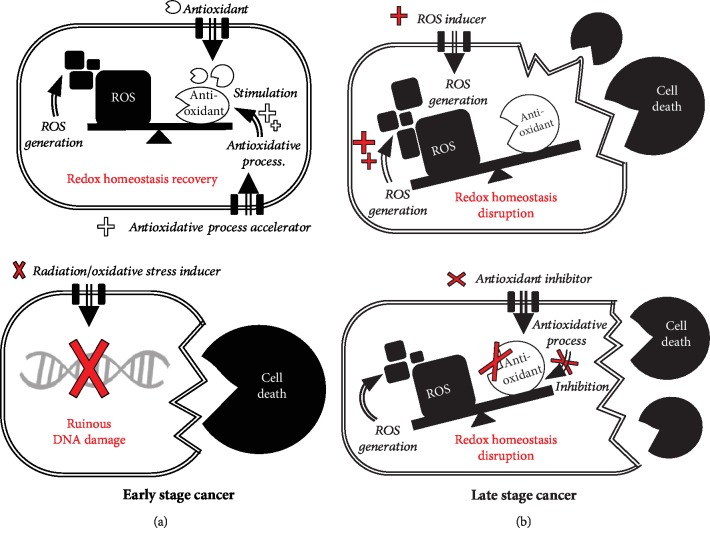
Anticancer therapeutic strategies attacking early-stage and late-stage cancer cells. (a) Early-stage cancer cells simply enable recovery of the disrupted redox status using antioxidants/antioxidative process accelerators. Briefly, chemotherapy with radiation or oxidative stress inducers is used to remove these cancer cells, in which significant DNA damage occurs. (b) Late-stage cancer cells have higher basal ROS levels and antioxidative activities than normal or early-stage cancer cells. In this case, cancer cells can be killed by redox homeostasis disruption following severe cytotoxic effects mediated by direct ROS inducers and/or antioxidant inhibitors. Prooxidative agents hold promise for potent cancer chemotherapy. The double-lined arrows and double-lined squares indicate the direction of anticancer molecules for movement and in cancer cells, respectively.

In the early stages, cancer cells exhibit uncontrolled cell growth and proliferation via the modulation of transcription factors and are vulnerable to DNA damage [56, 57] through therapeutic strategies focused on inducing genetic damage using radiation or oxidative stress [58–60] (Figure 2). In the late stages, metastatic cancers undergo metabolic changes such as increased endogenous antioxidant levels to buffer oxidative stress conditions [61]. Indeed, the GSH/GSSG ratio tends to be lower in circulating melanoma or metastatic cancers, suggesting that late-stage cancers have better antioxidant processes than early-stage cancers [62, 63]. Although NADPH-independent catalase activity has been reported to decrease with cancer progression [64], the remarkable antioxidant capacity is one of the reasons for chemoresistance in advanced cancer cells [65, 66]. ROS-inducing and/or antioxidant-suppressing approaches can be applied appropriately for the treatment of malignant cancer cells. Oxidative stress-modulated therapeutics for attacking cancer cells are being actively researched in the anticancer field [67, 68]. The cell-killing potential of ROS has been harnessed for anticancer therapies with two major approaches: direct ROS generation and antioxidant process inhibition [6].

### 3.1. Direct ROS Generation

Electrons derived from metabolism and respiratory processes are representative ROS sources in cells [69]. Impairing the respiratory cycles with the alteration of radical intermediates produces superoxide by which motexafin gadolinium and anthracyclines function [69–71]. Motexafin gadolinium, an avid electron acceptor, enhances the therapeutic index of radiotherapy, since it can inhibit the repair activities of cancer cells after irradiation [72, 73]. It is effective in patients with brain tumors, brain metastases, and pediatric gliomas [72]. Indeed, anthracycline-based anticancer drugs such as doxorubicin can induce the chelation of intracellular iron, leading to the accumulation of hydroxyl radicals and ultimately to cell death [74]. These drugs are effective for malignant lymphomas, acute leukemia, and diverse solid tumors [75]. Cisplatin, a well-known anticancer agent with cross-linking activity, directly damages mitochondrial DNA (mtDNA), which leads to ETC impairment [76]. It can also interfere with DNA replication and consequently induce oxidative stress to target cancer cells [77]. The drug is effective for diverse cancer types, especially ovarian cancer [78, 79]. 2-Methoxyestradiol is known to inhibit ETC complex I [80], inducing mitochondrial production of hydrogen peroxide [81]. Subsequently, it rapidly activates c-Jun N-terminal kinase (JNK), resulting in cytochrome c release and caspase-9 activation to initiate apoptosis [82, 83]. The drug can promote the therapeutic capability of other anticancer agents [84–86]. *In vitro* and *in vivo* studies have demonstrated that 2-methoxyestradiol-mediated chemotherapy can inhibit malignant cell proliferation as its own activity or in combination with synergistic drugs [87–90]. The ROS-accelerating anticancer agents described above are listed in Table 1.

Although anticancer drugs with direct ROS-accumulating activity have been shown to be effective for treating different types of cancer, the effects on normal cells are still controversial as they damage not only cancer cells but also normal cells. For instance, the radiosensitizer motexafin gadolinium interrupts the DNA repair process and causes injuries to surrounding normal cells [91]. Additionally, anthracyclines induce cardiotoxicity since their metabolites (e.g., oxygen-centered free radicals) can cause heart failure or cardiomyopathy, with a higher risk for younger patients [92–94]. Cisplatin-induced ototoxicity has been reported, attributed to its direct binding to DNA and consequent activation of the inflammatory cascade [95]. Additionally, liver function abnormalities, fatigue, and diarrhea have been reported in patients treated with 2-methoxyestradiol [85, 96, 97].

### 3.2. Antioxidant Process Inhibition

Although direct ROS induction is one of the effective strategies for treating malignant cancer cells [98], its combination with the disruption of antioxidative processes leads to the best results for overcoming the resistance characteristics of cancer cells. Depletion of GSH activity is regarded as an indirect method of generating oxidative stress. Cells can synthesize GSH via an ATP-dependent process catalyzed by glutamate-cysteine ligase (GCL) and GSH synthetase [99, 100]. For instance, buthionine sulfoximine, a typical GSH synthesis inhibitor, can bind to the GCL site that normally binds to the acceptor amino acid [101]. Imexon, a small-molecule chemotherapeutic agent, is widely used to treat advanced cancers of the breast, lung, and prostate. It can disrupt GSH activity by binding to the thiol functional group of reduced GSH [102, 103] and subsequently deplete the GSH pool for antioxidative activity. Due to a decrease in the GSH level by imexon treatment, loss of the mitochondrial membrane potential and the accumulation of oxidative stress occur in cancer cells.

Although anticancer therapy needs to disrupt, both directly and indirectly, the redox adaptation status of cancer cells, the inhibition of antioxidative enzyme has deleterious side effects on normal cells in tissues and organs. For instance, buthionine sulfoximine is known to be associated with cardiac hypertrophy and heart failure by inducing soluble epoxide hydrolase [104]. Imexon has potential side effects in normal cells due to its cytotoxicity [105–107]. For the future direction of oxidative stress-accelerating anticancer therapy, further study is needed to identify ways to not only reduce the side effects but also increase cancer cell-specific killing efficiency. For instance, cotreatment with antioxidant supplements that attenuate cisplatin-mediated nephrotoxicity through Nrf2 signaling has been investigated [108]. Moreover, plant-derived phytochemicals such as flavonoids and carotenoids that act as both antioxidants and prooxidants to improve the therapeutic effects and to reduce the cytotoxic effect have been reported [109–111].

## 4. Pathway Analysis to Understand the Process of Prooxidative Cancer Therapy

Identifying biological changes in cancer cells caused by anticancer drugs is meaningful to improve their therapeutic effect. Although several mechanism studies have been actively conducted to determine the mode of action of anticancer drugs for cancer treatment, the efficacy and toxicity of anti- and prooxidants remain controversial. In this regard, pathway analysis has the advantage of comprehensively elucidating the molecular network involved in the response to certain drugs. However, very few studies have been performed to explore biological modulation during treatment with prooxidant anticancer agents. In this review, we explore and visualize key information on drug-gene, drug-cell process, and drug-disease relationships for six anticancer drugs abovementioned with prooxidative activity (2-methoxyestradiol, buthionine sulfoximine, cisplatin, doxorubicin, imexon, and motexafin gadolinium) using a text mining-based biological network analysis tool, Pathway Studio ver. 12.2 (Elsevier, USA). This database provides information describing the relationships between the entities including the drugs, genes, cell processes, and diseases through a curated resource based on text mining from biology articles.

**Figure 3:**
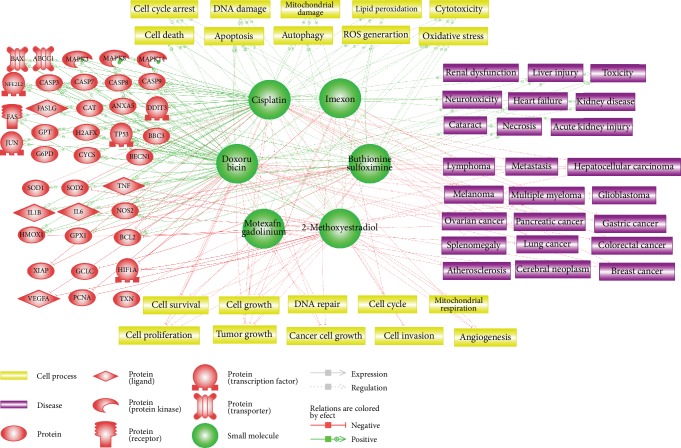
Proposed biological pathways related to prooxidative anticancer drugs. Comprehensive illustration of the drug-gene, drug-cell process, and drug-disease relationships for certain anticancer drugs with prooxidative activity (buthionine sulfoximine, cisplatin, doxorubicin, imexon, 2-methoxyestradiol, and motexafin gadolinium). Green and red lines denote the positive and negative effects of each drug, respectively. The legend for the diagrams is located at the bottom part of the figure. Target proteins (red), drug molecules (green), cell processes (yellow), and diseases (purple) are symbolized and organized in a complex biological network.

Each drug molecule was first inputted to build a network, and then the genes, cell processes, and diseases associated with the drugs were analyzed based on data provided in five or more references (Figure 3). Cisplatin and doxorubicin had the largest networks, implying that these two drugs have been extensively studied compared to the others, while imexon and motexafin gadolinium had the fewest connections. Figure 3 comprehensively illustrates the biological pathways including the target genes, key cellular processes, and target types of cancer that can be positively or negatively affected by these anticancer drugs. There were two types of relationships in the identified networks: Expression and Regulation. In Expression relationship, the drug alters the protein abundance by affecting the levels of transcript or protein stability. In Regulation relationships, the drug directly or indirectly changes the activity of the genes, cell processes, and diseases. In addition, we evaluated the possible side effects related to the prooxidant anticancer drugs such as neurotoxicity and cardiovascular diseases.  Table 2 summarizes the detailed information obtained from pathway analysis regarding the relationship of each drug with the targeted genes, cell processes, and diseases. We also explored the association of drug resistance with each drug through network analysis.

Based on the high number of references in the pathway analysis, we found that 2-methoxyestradiol is not only a potent inhibitor of HIF1A and VEGFA, which play important roles in angiogenesis [112], it also activates MAPK8, which triggers apoptosis [113]. Consistent with these results, 2-methoxyestradiol has been shown to be closely associated with cellular processes such as apoptosis, cell proliferation, and angiogenesis. Breast cancer, melanoma, and pancreatic cancer were predicted to be major targets for this drug, and atherosclerosis can also be attenuated due to its antiangiogenetic effects. Moreover, 2-methoxyestradiol-mediated autophagy promoting cancer cell survival could lead to drug resistance [114].

Buthionine sulfoximine was shown to effectively inhibit GCLC, blocking GSH synthesis [115]. The expression of GPX1 was also found to decrease while that of NFE2L2, HMOX1, and SOD2 increased in direct response to GSH depletion [116]. Oxidative stress, apoptosis, and cell death were identified as the main cell processes induced by buthionine sulfoximine-mediated GSH inhibition. Hepatocellular carcinoma and lung cancer were predicted to be the main target diseases, and cataract can be evoked by increased lipid peroxidation in the lens [117]. The increased NFE2L2 can upregulate ABCC1, which is a cell membrane transporter protein [118]. Accordingly, increased drug efflux through the transporter leads to drug resistance [119]. Buthionine sulfoximine-mediated autophagy can also negatively affect drug sensitivity.

Cisplatin was shown to significantly induce expression of the well-known tumor suppressor TP53 as well as proapoptotic genes such as TNF, BAX, CASP3, and FAS, while decreasing antiapoptotic BCL2 and XIAP expression. Consistently, cell processes including apoptosis, ROS generation, DNA damage, and mitochondrial damage were found to be significantly induced by cisplatin treatment. Diseases effectively targeted by cisplatin were predicted to be ovarian, lung, gastric, and breast cancer. However, cisplatin-induced proinflammatory cytokines IL1B, IL6, and TNF are at risk of causing side effects such as acute kidney injury and renal dysfunction. Cisplatin also plays important roles in drug resistance by inducing autophagy and activating NFE2L2 and ABCC1, which elevate drug efflux.

Doxorubicin was shown to have similar effects to cisplatin on targeted genes and cell processes. It also significantly increases TP53, BAX, TNF, CASP3, and FAS expression and decreases BCL2 and XIAP expression, promoting apoptosis. Oxidative stress, DNA damage, and lipid peroxidation were suggested to be doxorubicin-mediated cell processes. Doxorubicin is mainly used to treat breast, ovarian, and lung cancer as well as lymphoma, but there is a risk of heart failure and neurotoxicity. Drug resistance in doxorubicin was predicted to be attributable to increased autophagy and the upregulation of NFE2L2 and ABCC1.

Imexon was found to positively regulate the activity of CASP3 and CASP9 which have critical roles in apoptosis. Oxidative stress and cell cycle arrest can be stimulated by imexon, which was predicted to have therapeutic effects on multiple myeloma and splenomegaly.

Motexafin gadolinium was shown to inhibit the activity of TXN and HMOX1, leading to apoptosis. It was suggested to exhibit anticancer effects by promoting ROS generation and oxidative stress and by disrupting the DNA repair process. Motexafin gadolinium was expected to target diseases including lung cancer and cerebral neoplasm.

## 5. Conclusions

Redox homeostasis plays an essential role in maintaining diverse cellular processes [120]. The disruption of redox homeostasis is being actively investigated in the field of chemotherapy since cancer cells can be effectively killed by accelerating their oxidative stress state. In this review, we presented an overview of ROS-inducing anticancer therapy and the anticancer strategy using prooxidative agents in terms of direct and indirect ROS accumulation. For a comprehensive understanding of biological network of prooxidant drugs and molecular targets, our pathway analysis highlighted the crucial effects of each anticancer drug on genes, cell processes, and diseases related to ROS generation and antioxidant inhibition. Our explanation of changes in biological processes relevant to specific drugs and potential side effects would be meaningful for better understanding of the toxicological aspects as well as for predicting the efficacy of chemotherapies using prooxidative anticancer drugs with undetectable side effects. Although several previous studies have investigated the modes of action for prooxidant drugs, pharmacogenomic studies evaluating the drug treatments are still required to elucidate the exact anticancer mechanisms and potential molecular targets. Our review will help researchers better understand the current gene-targeting anticancer strategies involving prooxidative drugs in order to overcome their controversial side effects.

## Figures and Tables

**Table 1:** Mechanism of action of ROS-inducing anticancer drugs.

Name	Mechanism of action	Reference
*Direct ROS generation*		
Motexafin gadolinium	Accepts electrons to form superoxide	[69]
Doxorubicin	Induces chelation of iron to generate hydroxyl radical	[74]
Cisplatin	Damages mtDNA and ETC	[76]
2-Methoxyestradiol	Inhibits ETC complex I	[80]
*Antioxidant process inhibition*		
Buthionine sulfoximine	Binds to enzyme related to GSH synthesis	[101]
Imexon	Binds to thiol to GSH activity disruption	[102, 103]

**Table 2:** List of proteins, cell processes, and diseases targeted by anticancer drugs.

Drugs	Target type	Relation	Relation effect	Target
2-Methoxyestradiol	Protein	Expression	Positive	BAX, TP53
			Negative	HIF1A, IL6, PCNA, TNF, VEGFA
		Regulation	Positive	CASP9, MAPK8
			Negative	BCL2, HIF1A, SOD2
	Cell process	Regulation	Positive	Apoptosis, autophagy, cell cycle arrest, cell death, DNA damage, mitochondrial damage, oxidative stress, ROS generation
			Negative	Angiogenesis, cell cycle, cell growth, cell invasion, cell proliferation, cell survival, mitochondrial respiration, tumor growth
	Disease	Regulation	Negative	Atherosclerosis, breast cancer, hepatocellular carcinoma, melanoma, pancreatic cancer

Buthionine sulfoximine	Protein	Expression	Positive	BCL2, HMOX1, JUN, NFE2L2, SOD2, TNF
			Negative	GPX1, IL6, NOS2
		Regulation	Positive	BCL2, CASP3, MAPK14
			Negative	GCLC
	Cell process	Regulation	Positive	Apoptosis, autophagy, cell death, cytotoxicity, DNA damage, lipid peroxidation, oxidative stress, ROS generation
			Negative	Cell growth, cell proliferation, tumor growth
	Disease	Regulation	Positive	Cataract, liver injury, necrosis, neurotoxicity, toxicity
			Negative	Hepatocellular carcinoma, lung cancer

Cisplatin	Protein	Expression	Positive	ABCC1, BAX, BBC3, BECN1, CASP3, CASP8, CASP9, CYCS, DDIT3, FAS, FASLG, GPT, H2AFX, HMOX1, IL1B, IL6, JUN, NFE2L2, NOS2, TNF, TP53
			Negative	BCL2, SOD2, XIAP
		Regulation	Positive	CASP3, CASP7, CYCS, G6PD, MAPK14, MAPK3, MAPK8, TP53
			Negative	SOD1
	Cell process	Regulation	Positive	Apoptosis, autophagy, cell cycle arrest, cell death, cytotoxicity, DNA damage, lipid peroxidation, mitochondrial damage, oxidative stress, ROS generation
			Negative	Angiogenesis, cancer cell growth, cell growth, cell invasion, cell proliferation, cell survival, tumor growth
	Disease	Regulation	Positive	Acute kidney injury, kidney disease, liver injury, necrosis, neurotoxicity, renal dysfunction, toxicity
			Negative	Breast cancer, colorectal cancer, gastric cancer, hepatocellular carcinoma, lung cancer, lymphoma, melanoma, metastasis, ovarian cancer, pancreatic cancer

Doxorubicin	Protein	Expression	Positive	ABCC1, BAX, BBC3, BECN1, CASP3, CASP7, CASP8, CASP9, CAT, CYCS, DDIT3, FAS, FASLG, GPX1, H2AFX, HMOX1, IL1B, IL6, MAPK3, MAPK8, NFE2L2, NOS2, SOD1, TNF, TP53
			Negative	BCL2, PCNA, VEGFA, XIAP
		Regulation	Positive	ANXA5, CASP3, CASP7, CASP8, FAS, GPT, IL6, MAPK14, MAPK3, MAPK8, NOS2, TP53
			Negative	HIF1A
	Cell process	Regulation	Positive	Apoptosis, autophagy, cell cycle arrest, cell death, cytotoxicity, DNA damage, lipid peroxidation, mitochondrial damage, oxidative stress, ROS generation
			Negative	Angiogenesis, cancer cell growth, cell growth, cell proliferation, cell survival, DNA repair, mitochondrial respiration, tumor growth
	Disease	Regulation	Positive	Acute kidney injury, kidney disease, liver injury, necrosis, neurotoxicity, renal dysfunction, toxicity
			Negative	Breast cancer, colorectal cancer, gastric cancer, hepatocellular carcinoma, lung cancer, lymphoma, melanoma, metastasis, ovarian cancer, pancreatic cancer

Imexon	Protein	Expression	Negative	HIF1A
		Regulation	Positive	CASP3, CASP9
	Cell process	Regulation	Positive	Apoptosis, cell cycle arrest, oxidative stress
			Negative	Cancer cell growth, cell cycle, cell growth, tumor growth
	Disease	Regulation	Negative	Lymphoma, melanoma, multiple myeloma, splenomegaly

Motexafin gadolinium	Protein	Regulation	Negative	HMOX1, TXN
	Cell process	Regulation	Positive	Apoptosis, cell death, cytotoxicity, oxidative stress, ROS generation
			Negative	Cell proliferation, cell survival, DNA repair, tumor growth
	Disease	Regulation	Negative	Atherosclerosis, cerebral neoplasm, glioblastoma, lung cancer, metastasis
